# Association between cardiometabolic index and female infertility: A population-based study

**DOI:** 10.1371/journal.pone.0313576

**Published:** 2024-12-04

**Authors:** Lingxia Kong, Xian Ding, Qian Wang, Ruijie Xie, Fei Sun, Ningying Zhou, Chunting Li, Xiao Chen, Hong Qian

**Affiliations:** 1 Wuxi School of Medicine, Jiangnan University, Wuxi, China; 2 Department of Anesthesiology, Affiliated Hospital of Jiangnan University, Wuxi, China; 3 Department of Hand and Microsurgery, The Affiliated Nanhua Hospital, Hengyang Medical School, University of South China, Hengyang, China; 4 Department of Maternity Medicine, Affiliated Hospital of Jiangnan University, Wuxi, China; Bowen University, NIGERIA

## Abstract

**Background:**

One of the risk indicators of infertility is obesity. The cardiometabolic index (CMI) comprises obesity and blood lipids and is regarded as a novel indicator for evaluating obesity. Nevertheless, it is unclear whether it has any connection to infertility. This study set out to investigate the association between infertility and CMI.

**Methods:**

Based on cross-sectional data from the 2013–2018 National Health and Nutrition Examination Survey (NHANES), infertility and CMI statistics with complete information were selected. This study investigated the correlation between CMI and infertility using multivariate logistic regression analyses and subgroups. Use fitted smooth curves and threshold effect analysis to describe the nonlinear association between CMI and infertility.

**Results:**

202 (13.31%) among the 1720 participants that got involved in the investigation were female infertile. Among the three models, the outcomes confirmed a positive correlation between CMI levels and the incidence of infertility (OR = 1.12, 95% CI: 1.01–1.24). Additionally, significant relationships were maintained in subgroup analysis (p > 0.05). Smooth curve fitting indicated a nonlinear positive connection between CMI and infertility, and an inflection point of 0.93 (log-likelihood ratio *P* < 0.05) was shown by threshold effect analysis.

**Conclusion:**

Our findings suggest a significant relationship between CMI and infertility in American females. This helps identify high-risk groups for infertility, informing clinical practice and public health policy to improve metabolic and reproductive health.

## Introduction

The typical description of infertility is being unable to conceive after 12 months or more of regular, unprotected sexual behavior [1]. Infertility impacts over 1.86 million individuals globally, and in the United States, 8.8% of women between the age range of 15 and 49 suffer medical or psychological problems that render it harder for them to conceive naturally [2, 3]. Infertility encompasses much more than merely the loss of fertility; it also involves societal prejudice towards the patient and their family, gloom, pain, and low marital satisfaction [4]. Although the precise cause of infertility is unclear, there is mounting evidence that, in addition to pelvic tubal abnormalities and ovulation obstacles, the reasons for infertility are intimately linked to unhealthy lifestyles (such as obesity).

The obesity percentage among American women of reproductive age has risen to 37%. Obesity has emerged as a significant global public health concern [5]. Obese women who are of reproductive age are more inclined to encounter menstruation disorders, ovulation problems, decreased follicular growth and development, and infertility [6–8]. Due to aberrant lipid metabolism, obesity, a hallmark of metabolic diseases, could affect female fertility [7, 9]. The setting up precise metrics for lipid metabolism monitoring facilitates the timely detection of obesity. The body mass index (BMI), currently commonly employed to quantify generalized obesity, is less sensitive when assessing the degree of obesity [10]. The weight-adjusted waist index (WWI), which gauges the proportion of fat and muscle, is used to evaluate central obesity [11]. In 2015, Wakabayashi et al. introduced the cardiometabolic index (CMI), an innovative index that assesses the extent of obesity and lipid levels in individuals. The CMI may be used to determine the likelihood of metabolic syndrome in obese women [12–14]. Numerous studies have indicated that the risk of infertility may be identified by waist circumference (WC), body mass index (BMI), waist-to-hip ratio (WHI), and body size index (ABSI) [15–17]. Due to the limitations of BMI in reflecting the obesity phenotype [18–20], other obesity indicators have improved, but they lack the ability to reflect blood lipids. At present, the link between blood lipid levels and infertility remains uncertain. Studies have linked elevated LDL-C to an increased risk of female infertility, but the lack of studies comparing lipid-related characteristics with female infertility has limited the interpretation of the findings [21]. CMI can not only evaluate the degree of obesity, but also reflect the level of blood lipid metabolism, so CMI may be a better marker of the relationship between obesity, blood lipids and infertility.

Consequently, we investigated the relationship between CMI and infertility utilizing NHANES data.

## Methods

### Survey description and study population

The National Center for Health Statistics (NCHS) utilized the National Health and Nutrition Examination (NHANES) to compile a sample for this research to evaluate the nutritional status of American households. The public can view comprehensive NHANES demographic, nutritional, laboratory tests, physical examination, and questionnaire data. The public may access all NHANES study data at www.cdc.gov/nchs/nhanes/. It has an effectively representative sample, performing a nationwide stratified multistage probability sampling every two years. The NCHS Research Ethics Review Board approved all NHANES, and each participant completed informed consent forms. The NHANES database may be accessed without additional institutional review board approval.

**Fig 1:**
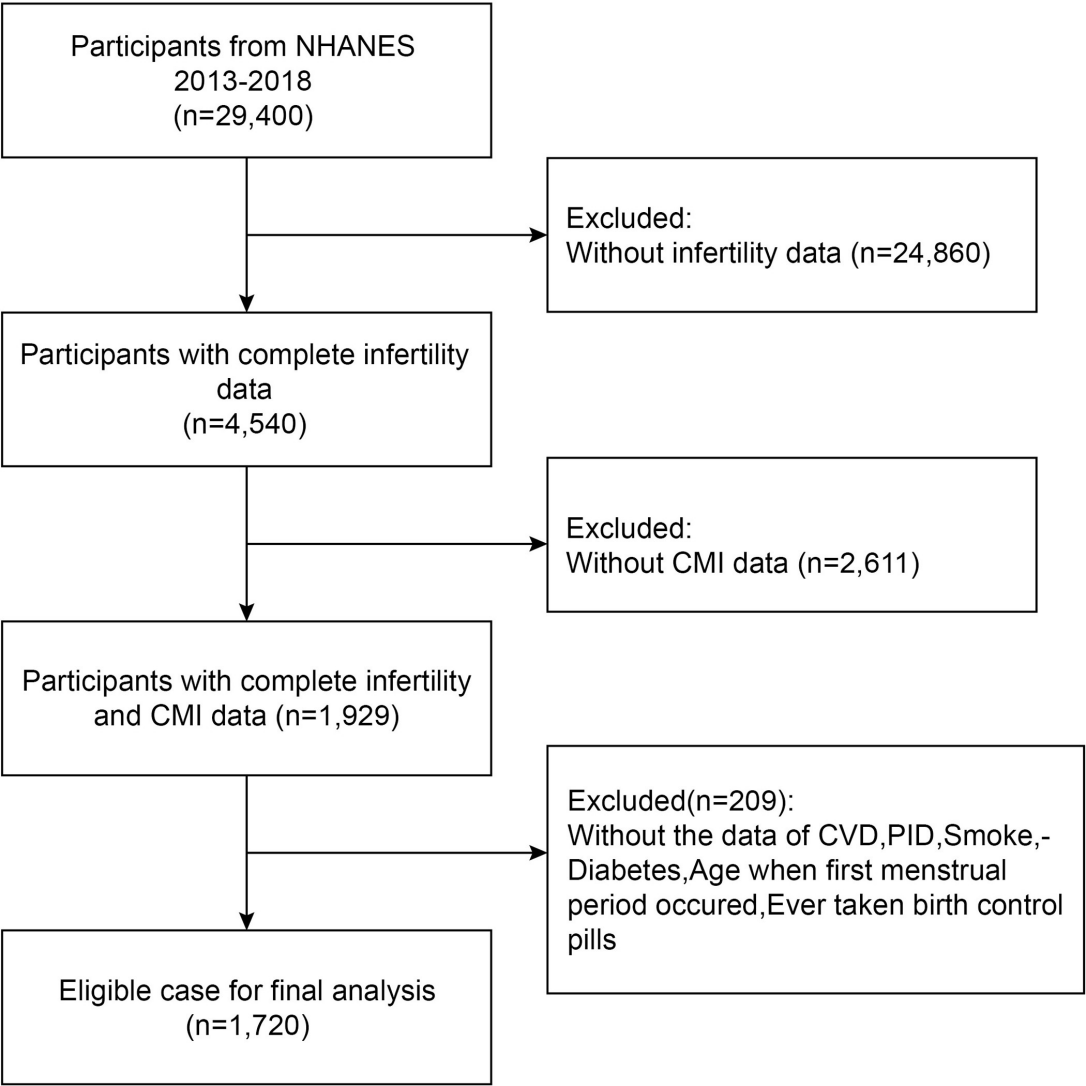
The flow chart displays the research populations included in the NHANES 2013–2018 data.

This research examined data from three cyclical NHANES from 2013 to 2018 (Fig 1), excluding minor (age <18) participants, and people with full CMI and infertility data were picked for inclusion. A total of 29,400 respondents were initially recruited. After eliminating those with missing self-reported infertility (n = 24,860), missing CMI data (n = 2611), and missing data on any of the factors in this research (n = 209), 1,720 suitable participants were subsequently included, ranging in age from 20 to 59.

### Cardiometabolic index and infertility definition

The outcome variable was infertility, and the Reproductive Health Questionnaire (RHQ) item RHQ074 supplied the data. "Have you ever attempted to get pregnant and been unsuccessful for at least a year?" Participants who answered "yes" were deemed infertile, whereas those who selected "no" were judged fertile. Therefore, the diagnosis of infertility is based on the participants’ self-reports.

CMI was intended to be used as an indicator of exposure. The data used for calculating CMI were obtained from measurements of waist circumference (WC), height, triglycerides (TG), and high-density lipoprotein cholesterol (HDL-C). The formula for computation is as follows: WHtR = WC(cm)/height(cm), CMI = WHtR ×[TG (mg/dL)/HDL-C (mg/dL)] [12].

### Covariates of interest

A variety of potential factors that might reinforce the link between CMI and infertility were also taken into account in this investigation. Demographics include, for instance, age (years), race (Mexican American/Other Hispanic/Non-Hispanic White/Non-Hispanic Black/Other Race), education level (below high school/high school/above high school), and marital status (married or living with a partner/living alone). The questionnaire obtained data on the following topics: age when first menstrual period occurred (<10/10 ≤ age <15/15 ≤ age ≤20), had regular periods in past 12 months (yes/no), ever taken birth control pills (yes/no), diabetes (yes/no), ever treated for a pelvic infection/PID(yes/no), smoked at least 100 cigarettes (yes/no), and cardiovascular disease (CVD). Self-reported questionnaires were utilized to assess cardiovascular disease. Any of the five criteria for coronary heart disease, angina, congestive heart failure, heart attack, or stroke indicates that participant have cardiovascular disease [22].

### Statistical analysis

Since NHANES follows the recommendations of the Centers for Disease Control and Prevention (CDC), it adopts a complex multi-stage sampling design. We compared two groups based on infertility status using t-tests for continuous variables and chi-square tests for categorical data. Continuous variables are expressed as standard deviation ± mean, whereas categorical data is displayed as frequencies and percentages. The association between CMI and infertility was explored using multivariable regression models, and three different logistic regression models were constructed for confounding factors. No variables were adjusted in Model 1. Model 2 was adjusted for age and race. Model 3 was adjusted for age, race, educational attainment, marital status, PIR, CVD, smoking, diabetes, age at first menstruation, regularity of menstruation during the previous 12 months, ever treated for a pelvic infection/PID, and ever taken birth control pills. We also converted these CMI into quartile categorical variables for sensitivity analysis to assess the stability of the results. Smoothed curve fitting was utilized to investigate the nonlinear relationship between CMI and infertility in this research. For the analysis of threshold effects, we compared the piecewise regression model with the singleton model by using a log-likelihood ratio test. Moreover, to testing the stability of the association between CMI and infertility, subgroup analyses of the relationship between CMI and infertility were performed using stratified multifactorial regression analysis. We stratified analysis for age, race (Mexican American/Other Hispanic/Non-Hispanic White/Non-Hispanic Black/Other Race), education level (below high school/high school/above high school), marital status (married or living with a partner/living alone), diabetes (yes/no), age when first menstrual period occurred (<10/10 ≤ age <15/15 ≤ age ≤20), had regular periods in past 12 months (yes/no), ever treated for a pelvic infection/PID (yes/no), ever taken birth control pills(yes/no), smoked at least 100 cigarettes (yes/no). In order to better reflect the distribution of the data and enhance the significance of differences between groups, we chose to use three-digit analysis. For the exact stratification of other covariates, please refer to the covariate selection mentioned above. PackageR (http://www.r-project.org) and EmpowerStats (http://www.enpowerstats.com) for all analyses. It was deemed statistically significant when *P* < 0.05.

## Results

### Baseline characteristics of participants

The demographic characteristics and other variables for each subject are arranged by infertility in Table 1. This research comprised 1720 individuals in total, with 202 women claiming self-reported infertility, making up 11.74% of the sample, with a mean age of 41.27 ± 10.90 years. Compared to fertile participants, women with infertility had higher family income and education levels, a higher prevalence of diabetes, a higher rate of receiving pelvic infection/PID treatment, and a lower prevalence of cardiovascular disease. Infertile women additionally possessed higher CMI (1.51 ± 2.00).

**Table 1:** Basic characteristics of the study participants in NHANES by infertility status.

Variables	Control	Infertility	*P*-value
	N = 1518	N = 202	
Age, years	40.00 ± 11.74	41.27 ± 10.90	0.155
PIR	2.43 ± 1.64	2.72 ± 1.63	0.013
Race, n (%)			0.382
Mexican American	264 (17.39%)	27 (13.37%)	
Other Hispanic	179 (11.79%)	21 (10.40%)	
Non-Hispanic White	493 (32.48%)	78 (38.61%)	
Non-Hispanic Black	324 (21.34%)	44 (21.78%)	
Other Race	258 (17.00%)	32 (15.84%)	
Education level, n (%)			0.011
Less than high school	107 (7.05%)	6 (2.97%)	
High school	468 (30.83%)	51 (25.25%)	
More than high school	943 (62.12%)	145 (71.78%)	
Marital Status, n (%)			
Married or living with a partner	856 (56.39%)	153 (75.74%)	
Living alone	662 (43.61%)	49 (24.26%)	
Diabetes, n (%)			0.026
Yes	124 (8.17%)	26 (12.87%)	
No	1394 (91.83%)	176 (87.13%)	
Age when first menstrual period occurred, n (%)			0.486
Age<10	71 (4.68%)	12 (5.94%)	
10≤Age<15	1228 (80.90%)	166 (82.18%)	
15≤Age≤20	219 (14.43%)	24 (11.88%)	
Had regular periods in past 12 months, n (%)			0.918
Yes	1020 (67.19%)	135 (66.83%)	
No	498 (32.81%)	67 (33.17%)	
Ever treated for a pelvic infection/PID, n (%)			<0.001
Yes	79 (5.20%)	26 (12.87%)	
No	1439 (94.80%)	176 (87.13%)	
Ever taken birth control pills, n (%)			0.261
Yes	1038 (68.38%)	146 (72.28%)	
No	480 (31.62%)	56 (27.72%)	
Smoked at least 100 cigarettes, n (%)			0.251
Yes	502 (33.07%)	75 (37.13%)	
No	1016 (66.93%)	127 (62.87%)	
CVD, n (%)			0.012
Yes	74 (4.87%)	2 (0.99%)	
No	1444 (95.13%)	200 (99.01%)	
CMI	1.27 ± 1.29	1.51 ± 2.00	0.023

CMI, cardiometabolic index; PIR, poverty income ratio; CVD, cardiovascular disease.

### Association of cardiometabolic index with infertility

The association between CMI and infertility is demonstrated in Table 2. According to the outcome of the logistic regression study, there was a positive correlation between CMI and infertility in both model 1 and model 2. The positive relationship was also steady in the fully rectified model 3 (OR = 1.12, 95% CI:1.01–1.24, *P* = 0.0330). We observed that for every unit, an increase in CMI was associated with a 12% increased risk of infertility. Our further analyses, adjusting the continuous variable of CMI into categorical variables (quartiles), still showed statistically significant results. For each unit increase in CMI, participants in the highest quartile of CMI had an 88% higher prevalence of infertility than participants in the lowest quartile of CMI (OR = 1.88, 95% CI: 1.12–3.15, *p* for trend = 0.0593).

**Table 2:** Associations between cardiometabolic index and the risk of infertility.

Exposure	Model 1[OR (95%CI)]	Model 2[OR (95%CI)]	Model 3[OR (95%CI)]
CMI (continuous)	1.10 (1.01, 1.21)	1.10 (1.01, 1.21)	1.12 (1.01, 1.24)
	0.0252	0.0298	0.0330
CMI (quartile)			
Quartile 1 (n=430)	Reference (1)	Reference (1)	Reference (1)
Quartile 2 (n=430)	1.48 (0.94, 2.34)	1.51 (0.95, 2.38)	1.52 (0.93, 2.48)
	0.0896	0.0801	0.0965
Quartile 3 (n=430)	1.84 (1.18, 2.86)	1.89 (1.21, 2.97)	2.16 (1.33, 3.51)
	0.0071	0.0055	0.0018
Quartile 4 (n=430)	1.64 (1.04, 2.57)	1.66 (1.04, 2.65)	1.88 (1.12, 3.15)
	0.0316	0.0341	0.0168
*P* for trend	0.1019	0.1212	0.0593

Model 1: No covariates were adjusted.

Model 2: Adjusted for age and race.

Model 3: Adjusted for age, race, ratio of family income to poverty, education level, marital status, diabetes, cardiovascular disease history, smoked at least 100 cigarettes, age when first menstrual period occurred, ever taken birth control pills, ever treated for a pelvic infection/PID, had regular periods in past 12 months.

**Fig 2:**
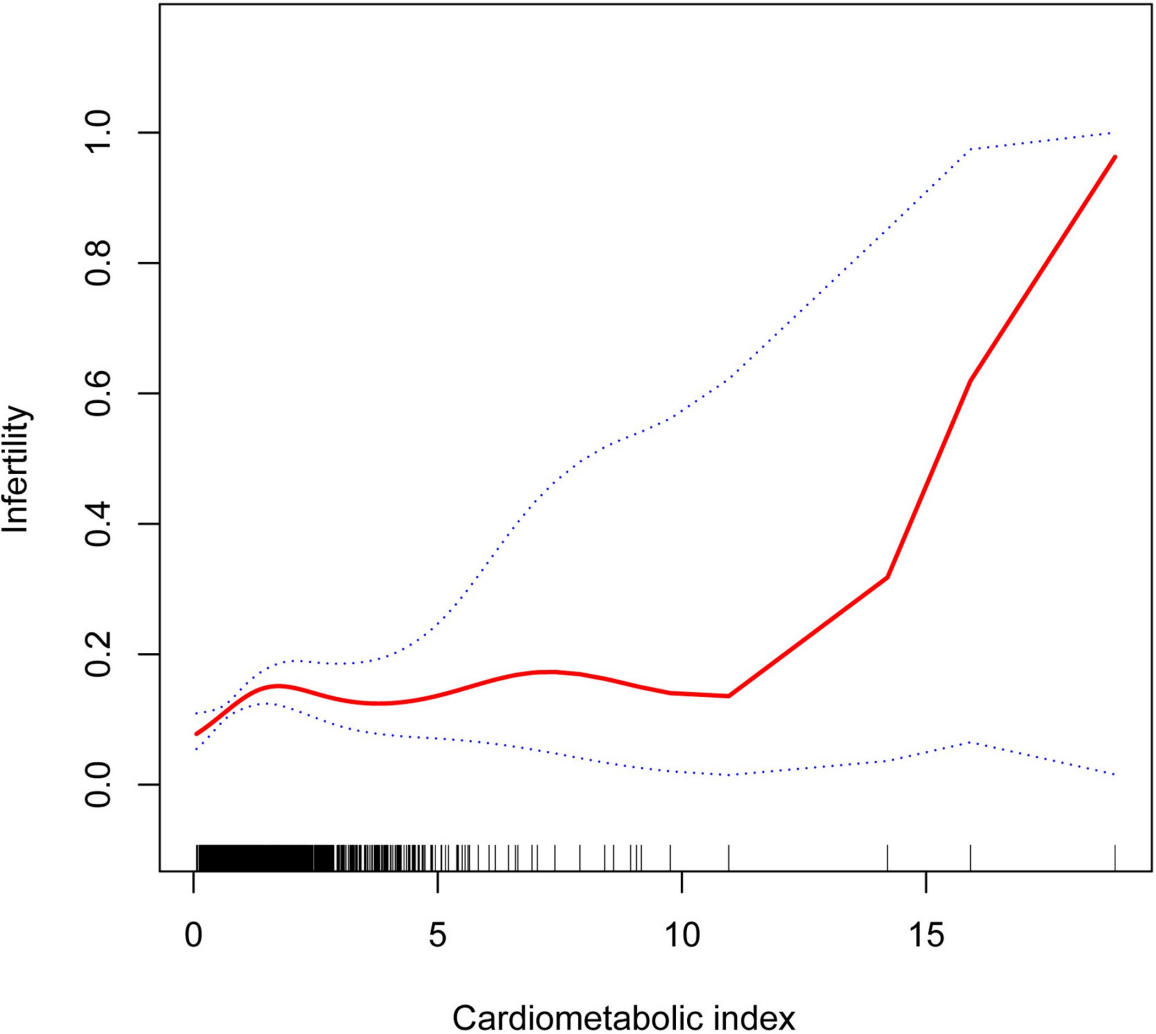
Smooth curve fitting for CMI and infertility.

We analyzed further the nonlinear correlation between CMI and infertility using smooth curve fitting. The results indicated a positive nonlinear relationship between CMI and infertility (Fig 2). We applied breakpoint analysis to determine the inflection point of the nonlinear relationship. The analysis revealed that the inflection point was 0.93 (log-likelihood ratio *P* < 0.05). When the CMI was under 0.93, the odds of infertility prevalence increased by 162% for every unit increase in CMI (OR = 2.62,95% CI:1.27–5.44, *p* = 0.0094). When CMI was more than 0.93 (OR = 1.06,95% CI: 0.94–1.18, *p* = 0.3468), there was no correlation with the odds of infertility prevalence (Table 3). We further analyzed the potential relationship between CMI and infertility in non-obese women. The results showed no association between CMI and infertility in people with a BMI < 30 (S1 Table). In addition, we also performed multiple logistic regression on the relationship between BMI and infertility. (S2 Table)

**Table 3:** The threshold effect analysis of cardiometabolic index on infertility was analyzed using a 2-piecewise linear regression model before and after adjusting for covariates.

	Model 1	Model 3
	OR (95%CI), *P*-value	OR (95%CI), *P*-value
Fitting by the standard linear model	1.10 (1.01, 1.21) 0.0252	1.12 (1.01, 1.24) 0.0330
Fitting by the 2-piecewise linear model		
Inflection point	0.71	0.93
< K segment effect	3.24 (1.15, 9.14) 0.0264	2.62 (1.27, 5.44) 0.0094
> K segment effect	1.06 (0.96, 1.17) 0.2722	1.06 (0.94, 1.18) 0.3468
Log likelihood ratio	0.035	0.018

Model 1: No covariates were adjusted.

Model 3: Adjusted for age, race, ratio of family income to poverty, education level, marital status, diabetes, cardiovascular disease history, smoked at least 100 cigarettes, age when first menstrual period occurred, ever taken birth control pills, ever treated for a pelvic infection/PID, had regular periods in past 12 months.

### Subgroup analysis

To assess if the link between CMI and infertility was stable in the presence of the effect of other variables, we conducted subgroup analyses according to age, race, education level, marital status, diabetes, smoking, age at first menstruation, regularity of menstruation in the past 12 months, ever treated for a pelvic infection/PID, and ever taken birth control pills. None of these factors impacted the positive association between CMI and infertility, as shown in Table 4. Our study observed that individuals in the lowest age tertile had 33% higher odds of infertility prevalence for every unit rise in CMI (OR = 1.33,95% CI:1.06–1.68). When diabetes is absent, there is a 16% higher odds of infertility prevalence for every unit increase in CMI (OR = 1.16, 95% CI: 1.02–1.32). The onset menstruation age was 10 to 15 years old; with every unit rise in CMI, the prevalence of infertility rose by 14% (OR = 1.14,95% CI: 1.02–1.28). For every unit increase in CMI, the odds of infertility prevalence rose by 17% in patients who smoked (OR = 1.17,95% CI:1.02–1.33). In addition, we performed a subgroup analysis of BMI, but the results were not significant (S3 Table).

**Table 4:** Subgroup analyses of the effect of CMI on infertility.

Subgroups	N		OR (95% CI), *P*-value	*P* for interaction
	Total	Infertility		
Age				0.2174
Tertile 1	566	54 (26.73%)	1.33 (1.06, 1.68) 0.0147	
Tertile 2	557	83 (41.09%)	1.06 (0.91, 1.24) 0.4699	
Tertile 3	597	65 (32.18%)	1.07 (0.85, 1.34) 0.5583	
Race				0.8735
Mexican American	291	27 (13.37%)	1.20 (0.92, 1.55) 0.1787	
Other Hispanic	200	21 (10.40%)	0.94 (0.57, 1.55) 0.7960	
Non-Hispanic White	571	78 (38.61%)	1.14 (0.97, 1.35) 0.1065	
Non-Hispanic Black	368	44 (21.78%)	1.17 (0.78, 1.75) 0.4440	
Other Race	290	32 (15.84%)	1.00 (0.66, 1.49) 0.9833	
Education lever				0.9454
Less than high school	113	6 (2.97%)	1.03 (0.28, 3.87) 0.9625	
High school	519	51 (25.25%)	1.10 (0.94, 1.29) 0.2254	
More than high school	1088	145 (71.78%)	1.14 (0.99, 1.31) 0.0639	
Marital Status				0.7955
Married or living with a partner	1009	153 (75.74%)	1.13 (1.00, 1.28) 0.0563	
Living alone	711	49 (24.26%)	1.10 (0.91, 1.32) 0.3190	
Diabetes				0.4225
Yes	150	26 (12.87%)	1.06 (0.87, 1.29) 0.5867	
No	1570	176 (87.13%)	1.16 (1.02, 1.32) 0.0192	
Age when first menstrual period occurred				0.1264
Age<10	83	12 (5.94%)	0.66 (0.34, 1.26) 0.2056	
10≤Age<15	1394	166 (82.18%)	1.14 (1.02, 1.28) 0.0202	
15≤Age≤20	243	24 (11.88%)	1.29 (0.91, 1.83) 0.1535	
Had regular periods in past 12 months				0.4981
Yes	1155	135 (66.83%)	1.09 (0.97, 1.23) 0.1575	
No	565	67 (33.17%)	1.18 (0.96, 1.45) 0.1109	
Ever treated for a pelvic infection/PID				0.3677
Yes	105	26 (12.87%)	1.29 (0.92, 1.82) 0.1409	
No	1615	176 (87.13%)	1.11 (0.99, 1.24) 0.0829	
Ever taken birth control pills				0.9828
Yes	1184	146 (72.28%)	1.11 (0.99, 1.25) 0.0785	
No	536	56 (27.72%)	1.12 (0.93, 1.34) 0.2457	
Smoked at least 100 cigarettes				0.3296
Yes	577	75 (37.13%)	1.17 (1.02, 1.33) 0.0211	
No	1143	127 (62.87%)	1.06 (0.89, 1.25) 0.5338	

CMI, cardiometabolic index; OR, odd ratio; CI, confidence interval.

## Discussion

We observed a positive correlation between CMI and infertility in this cross-sectional research with 1720 participants, indicating that a higher CMI may raise the odds of infertility prevalence. And the subgroup analysis showed that this connection did not change. Moreover, there was a positive correlation between CMI levels and infertility when the CMI was less than 0.93. The relationship between CMI and infertility was not statistically significant when CMI was higher than 0.93. According to the research, controlling CMI levels may help manage infertility prevalence. CMI is a novel metric for assessing visceral fat distribution and dysfunction, and it has applications in predicting diabetes, hypertension, related cardiovascular metabolic conditions, and other diseases [23–25]. As far as we know, this is the first research to examine the relationship between CMI and the probability of being infertile.

While numerous studies have investigated the relationship between obesity and infertility, results have been inconsistent. A recent systematic evaluation and meta-analysis of the influence of lifestyle interventions before pregnancy on reproductive outcomes revealed that these interventions resulted in improved fat reduction and increased rates of successful pregnancies and live births [26]. Separate research showed that lifestyle modifications had a beneficial effect on the sexual functioning, cardiovascular well-being, and fertility of obese women who were experiencing infertility [27]. Interestingly, Legro et al. investigated a 16-week preconception lifestyle intervention on 379 obese infertile women. The findings of this randomized controlled trial indicate that women in the intervention group went through a 7 percent reduction in body weight on average and showed improvements in cardiometabolic markers compared to the control group. However, the intensive preconception lifestyle intervention did not enhance pregnancy rates, live births, length of pregnancy, or infant birth weight [28]. Similarly, a randomized controlled research study in Sweden had comparable findings. The trial included a 12-week weight-reduction intervention before conception. However, the trial did not demonstrate a substantial effect on live birth rates in women undergoing IVF compared to the group that did not undergo weight loss [29].

Presently, the prevailing method for evaluating the extent of obesity involves measuring BMI and waist circumference. Recently, several researchers have investigated the use of WWI as a measure of obesity. Ying et al. discovered a positive correlation between rising waist circumference and a higher probability of suffering infertility [15]. Furthermore, Wen & Zhong et al. observed that WWI was significantly and positively associated with infertility risk [17, 30]. Similarly, our research shows that an elevation in CMI leads to a more significant prevalence of infertility. The degree of obesity and lipid levels tend to affect fertility. CMI reflects the distribution of visceral adipose tissue and blood lipid levels [31]. CMI may become a new obesity indicator to predict the incidence of infertility. Apart from early recognition of infertility, CMI has also been linked to several other illnesses. Song et al. found a positive correlation between CMI and higher levels of insulin resistance (IR: OR = 3.46), impaired fasting glucose (IFG: OR = 1.27), and type 2 diabetes mellitus (T2DM: OR = 2.01). They also suggested that CMI has the potential to be used as a simple and reliable metabolic assessment [32]. Lazzer et al. demonstrated that CMI and visceral obesity index (VAI) exhibited higher sensitivity and specificity in evaluating metabolic syndrome in obese women, as compared to other obesity indices such as waist-to-hip ratio (WHR), body fat index (BMFI), and WHtR [33].

Multiple studies have shown a strong correlation between obesity and fertility. Typically, Generally, obese women are more likely to experience irregular menstrual cycles, ovulatory disorders, and infertility [34]. The regular functioning of women’s menstrual cycle and ovulation is regulated by the hypothalamic-pituitary-ovarian (HPO) axis. Obesity affects the balance of the HPO axis primarily by increasing leptin levels [35, 36]. Increased levels of leptin interfere with the release of gonadotropin-releasing hormone (GnRH) in the hypothalamus, causing irregular production of luteinizing hormone (LH) and follicle-stimulating hormone (FSH). This disruption affects the menstrual cycle and ovulation capacity [37]. Obesity decreases the production of adiponectin, a hormone that has anti-inflammatory and insulin-sensitizing characteristics [38]. Reduced adiponectin levels may result in insulin resistance, leading ovarian cells to overproduce androgens. This excessive production of androgens affects the secretion of GnRH, FSH, and LH, disrupting the HPO axis [39, 40]. Furthermore, excess fatty tissue promotes inflammation and oxidative stress, decreasing the quality of oocytes and uterine tolerance [41, 42]. CMI is the product of WHtR and TG/HDL-C. WHtR reflects the amount of fat accumulated under the skin. Research has shown that higher WHtR values are associated with decreased fertility [16]. TG/HDL-C serves as an indicator of metabolic condition. Lipid metabolism disorders affect cardiovascular well-being and raise levels of reactive oxygen species (ROS) due to excessive lipid accumulation, resulting in oxidative stress and ovarian granulosa cell stress that can damage the reproductive system [43–45].

Additionally, studies have shown that women who are obese are predisposed to developing polycystic ovarian syndrome (PCOS), which may be attributed to the fact that obesity induces obstruction of ovulation and excessive levels of male hormones in those with PCOS, causing metabolic irregularities and decreased fertility [46, 47]. To enhance their fertility, most women unable to conceive may make lifestyle alterations, including changes to their food, engaging in fitness programs, and making psychological modifications. Also, there is a significant number of individuals who actively use Assisted Reproductive Technology (ART) as a means of achieving pregnancy [48]. Nevertheless, the disruption of oocyte maturation and impaired spindle morphology in obese women significantly contribute to the higher rates of failure in ART procedures for this group [49, 50].

In conclusion, our study presents several advantages. The data in this research were derived from the NHANES, making the results applicable to the U.S. As far as we are aware, this is the first research to look at the relationship between CMI and the occurrence of infertility while considering the effects of obesity and metabolic profiles on infertility. Moreover, we accounted for pertinent confounders to more precisely evaluate the dependability of the correlation between CMI levels and infertility. Nevertheless, it is essential to admit certain limitations of our research. First, this cross-sectional research only examined the correlation between CMI and the likelihood of infertility prevalence. The aim was to provide some reference values for the early identification of infertility occurrence. However, the study was unable to demonstrate a causal link between CMI and infertility. Second, our findings are limited to specific country regions, and it is yet to be determined if they are to populations in different nations. In addition, despite the inclusion of some potential covariates in this research, other variables may still impact the likelihood of experiencing infertility, such as the shortage of diagnostic information about PCOS. Finally, the diagnosis of infertility relies on self-reported data, which unavoidably involves some recall bias caused by the potential for inaccurate recollection.

## Conclusion

Our findings suggest a significant relationship between CMI and infertility in American females. Levels of CMI might be a meaningful indicator of infertility. However, we still need further extensive prospective research to authenticate the findings of this research. This helps identify high-risk groups for infertility, informing clinical practice and public health policy to improve metabolic and reproductive health.

## Supporting information

S1 Table Association between CMI and infertility stratified by BMI < 30. (DOCX)

S2 Table Associations between BMI and infertility. (DOCX)

S3 Table Association between CMI and infertility stratified by BMI. (DOCX)
