# Supplementary material for: Association between cardiometabolic index and female infertility: A population-based study
Source: PLoS One. 2024 Dec 4;19(12):e0313576. doi: 10.1371/journal.pone.0313576 (PMC11616860; doi:10.1371/journal.pone.0313576)
Supplement: S2 Table — (DOCX) [file pone.0313576.s002.docx]

S2 Table Associations between BMI and infertility

| **Exposure** | **Model 1[OR (95%CI)]** | **Model 2[OR (95%CI)]** | **Model 3[OR (95%CI)]** |
| --- | --- | --- | --- |
| BMI ＜25 | 1.05 (0.91,1.21) | 1.01(0.87,1.17) | 1.02 (0.85,1.22) |
| *P* value | 0.5444 | 0.9376 | 0.8301 |
| 25≤BMI＜30 | 1.05 (0.84,1.32) | 1.04 (0.83,1.31) | 1.07 (0.83,1.39) |
| *P* value | 0.6610 | 0.7289 | 0.5976 |
| BMI ≥30 | 1.00 (0.97,1.03) | 1.00 (0.97,1.03) | 1.00 (0.96,1.04) |
| *P* value | 0.9682 | 0.9868 | 0.9316 |
| Model 1: No covariates were adjusted.  Model 2: Adjusted for age and race.  Model 3: Adjusted for age, race, ratio of family income to poverty, education level, marital status, diabetes, cardiovascular disease history, smoked at least 100 cigarettes, age when first menstrual period occurred, ever taken birth control pills, ever treated for a pelvic infection/PID, had regular periods in past 12 months. | | | |
